# Clinicopathologic Study of 28 Cases of Tubulocystic Renal Cell Carcinoma: Is It Time to Reclassify It as a Tubulocystic Renal Cell Tumor? 

**DOI:** 10.7759/cureus.74015

**Published:** 2024-11-19

**Authors:** Guang-Qian Xiao, Vidyadhari Karne, Stephen Phan, Alicia Cuber, Richard Chiu, William D Wallace

**Affiliations:** 1 Pathology, University of Southern California Keck School of Medicine, Los Angeles, USA

**Keywords:** carcinoma, classification, end-stage kidney disease, immunohistochemistry, renal cell, tubulocystic, tubulocystic renal cell carcinoma, tumor

## Abstract

Tubulocystic renal cell carcinoma (TC-RCC) is uncommon and is defined by exclusive tubulocystic growth. Its clinicopathology is still evolving. Twenty-eight cases of so-defined TC-RCC were studied for clinicopathology as well as, in some cases, immunohistochemistry. The study showed the tumor had a male predominance; tumor size ranged from 0.1 cm to 3.5 cm; multifocality and peripheral location were common. More than 1/3^rd^ of the cases presented with pseudoinvasion into fat; 54% of cases concurred with papillary adenoma(s); 53% of cases concurred with at least one other type of low-grade/indolent renal cell carcinoma. Background kidney displayed end-stage kidney disease in 96% of the cases. The tumor was positive for AMACR, negative for GATA3, and rarely/focally positive or completely negative for CK7 and CAIX. All presented with benign clinical courses. Given its frequent association with end-stage kidney disease and other indolent renal neoplasms as well as its uneventful clinical course, we proposed to reclassify it as a tubulocystic renal cell tumor.

## Introduction

Tubulocystic renal cell carcinoma (TC-RCC) is a rare cystic epithelial neoplasm with indolent clinical behavior [1]. It is newly defined by the WHO as a renal tumor with exclusive tubulocystic growth [2]. Our knowledge of this tumor is still evolving. In this study, we aimed to explore its clinicopathologic and immunohistochemical (IHC) features, and, given its common association with end-stage kidney disease as well as a benign clinical course, we proposed to reclassify it as a tubulocystic renal cell tumor.

## Materials and methods

Tubulocystic renal cell carcinoma was defined according to the International Society of Urologic Pathologists (ISUP) and WHO criteria [2,3]. Only cases with a pure tubulocystic pattern confirmed by examining the entire tumor from nephrectomy specimens were included in this study. Tumors with a mixture of tubulocystic and other patterns were excluded. So-defined TC-RCC cases accessioned at our institution, University of Southern California Keck School of Medicine, Los Angeles, CA, USA, from 2016 to mid-2024 were retrieved and reviewed for pathologic parameters, including tumor location, tumor size, background kidney, other concurrent types of renal cell tumor, and IHC profile when available. Patients’ demographics and clinical follow-up data were also recorded. A total of 28 so-defined TC-RCC cases were found and analyzed.

## Results

**Figure 1:**
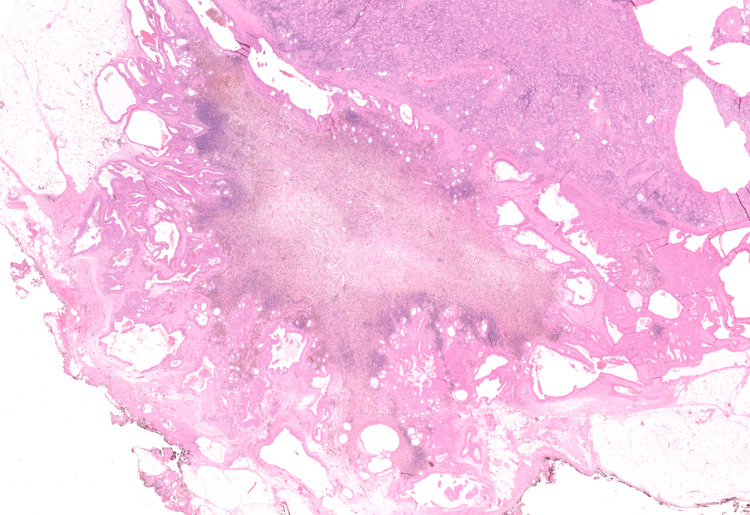
Macroscopic view of the tubulocystic tumor in the perinephric fat with a central scar from a previous biopsy

Patients' ages ranged from 23 to 85 (53±14) years with a male-to-female ratio of 8:1 and a left-kidney-to-right kidney ratio of 4:3. Tumor size ranged from 0.1 cm to 3.5 cm (1.2±0.8). Multifocality (≥2 foci) was common (68%, 19/28) (Table 1). The vast majority (93%, 26/28) were located at the periphery of renal parenchyma or near the renal pelvis, and most (75%, 21/28) were seen in the medulla or corticomedullary junction. Ten cases (36%) presented with protrusion into fat or pseudoinvasion into fat (Figure 1).

**Table 1:** Clinicopathology and immunohistochemistry of the 28 TC-RCC cases M: male; F: female; TC-RCC: tubulocystic renal cell carcinoma; cRCC: clear cell renal cell carcinoma; pRCC: papillary renal cell carcinoma; cPRCT: clear cell (tubule)papillary renal tumor; ACD-RCC: acquired cystic disease-associated renal cell carcinoma; FH: fumarate hydratase; yrs: years

Age (yrs)/gender	Laterality	Medulla (M) cortex (C)	Location of the tumor	Size (largest)	Focality	Papillary adenoma	Non-tumor kidney	Concurrent RCC	Immunohistochemistry	Follow-up data
64/M	Left	M/C	Non- peripheral	1.3 cm	3	Present	End-stage	Absent	AMACR+, Ker903-, P63-, rare CK7+, CD10+	No metastases or death (8 yrs)
42/M	Right	M	Peripheral	1.2 cm	2	Absent	End-stage	pRCC	AMACR+, TFE3-, CD10+, very focal CK7+	No metastases or death (6 yrs)
64/M	Left	M	Peripheral	1.0 cm	1	Absent	End-stage	cPRCT,cRCC	Not performed	No metastases or death (6 yrs)
62/M	Right	M/C	Peripheral	1.6 cm	1	Absent	End-stage	Absent	AMACR+, rare CAIX+, focal CK7+	No metastases or death (6 yrs)
66/M	Right	M	Peripheral	1.0 cm	1	Present	End-stage	ACD-RCC	AMACR+, BAP1+, CAIX- very focal CK7+	No metastases or death (6 yrs)
59/M	Left	C	Peripheral	1.7 cm	1	Absent	End-stage	cRCC	Not performed	No metastases or death
85/M	Right	M/C	Peripheral	3.5 cm	2	Absent	End-stage	Absent	Not performed	Not available
33/M	Left	M	Peripheral	0.5 cm	2	Absent	End-stage	Absent	Not performed	No metastases or death (4 yrs)
50/M	Right	M/C	Peripheral	0.7 cm	5	Present	End-stage	cRCC	AMACR+, CAIX-, p63-, rare CK7+	No metastases or death (3 yrs)
53/F	Right	C	Peripheral	1.8 cm	1	Present	End-stage	Absent	AMACR+, CAIX-, P63-, focal CK7+	No metastases or death (3 yrs)
47/M	Left	M	Peripheral	0.5 cm	1	Present	End-stage	ACD-RCC	Not performed	No metastases or death (2 yrs)
57/M	Left	M/C	Peripheral	0.8 cm	3	Present	Non-end stage	pRCC	Not performed	No metastases or death (2 yrs)
72/M	Right	C	Peripheral	0.2 cm	2	Present	End-stage	ACD-RCC, pRCC	Not performed	No metastases or death (3 yrs)
51/F	Left	M	Peripheral	2.4 cm	2	Absent	End-stage	Absent	AMACR+, Cathepsin K-, PAX8+, CD117-, GATA3-, CK7-, Ki67<1%	No metastases or death (2 yrs)
33/M	Left	M	Peripheral	0.5 cm	1	Present	End-stage	ACD-RCC, cPRCT	AMACR+, CAIX-, rare CK7+	No metastases or death 2 yrs)
36/F	Left	M	Peripheral	0.4 cm	2	Absent	End-stage	Absent	AMACR+, CAIX-, very focal CK7+	No metastases or death (2 yrs)
49/M	Left	M	Peripheral	0.5 cm	2	Present	End-stage	ACD-RCC	Not performed	No metastases or death (2 yrs)
68/M	Right	M/C	Non-peripheral	0.4 cm	1	Present	End-stage	Absent	Not performed	No metastases or death (2 yrs)
53/M	Right	C	Peripheral	0.3 cm	3	Absent	End-stage	ACD-RCC	Not performed	No metastases or death (1 yr)
53/M	Left	C	Peripheral	0.9 cm	2	Absent	End-stage	ACD-RCC	Not performed	No metastases or death (1 yr)
47/M	Left	C	Peripheral	0.5 mm	3	Present	End-stage	Absent	Not performed	No metastases or death (1 yr)
23/M	Left	M/C	Peripheral	0.3 mm	5	Absent	End-stage	Absent	Not performed	No metastases or death (1 yr)
65/M	Left	M	Peripheral	0.5 cm	1	Present	End-stage	cRCC	AMACR+, CAIX-, rare CK7+	No metastases or death (1 yr)
58/M	Left	M	Peripheral	1.0 cm	2	Absent	End-stage	cRCC	AMACR+, CAIX-, FH+, GATA3-, very focal CK7+,	No metastases or death (1/2 yr)
66/M	Right	C	peripheral	1.6 cm	2	Present	End-stage	Absent	AMACR+, CAIX-, GATA3-, Brst2-, AR+, focal CK7+	No metastases or death (3 yrs)
59/M	Right	M/C	peripheral	1.0 cm	2	Present	End-stage	Absent	Not performed	No metastases or death (1/2 yr)
74/M	Left	M/C	Peripheral	0.7 mm	2	Present	End-stage	cRCC, ADC-RCC	Not performed	No metastases or death (2 yrs)
55/M	Right	M, M/C	Peripheral	1.2 cm	3	Absent	End-stage	Atypical renal cysts	AMACR+, CAIX-, GATA3-, Brst2-, AR+, focal CK7+,	No metastases or death (1/2 yr)

Concurrent other types of renal cell tumor

Among the 28 cases of TC-RCC, 15 (54%) concurred with papillary adenoma, five (18%) concurred with acquired cystic disease-associated renal cell carcinoma (ACD-RCC), four (14%) concurred with clear cell renal cell carcinoma (cRCC), two (7%) concurred with papillary renal cell carcinoma (pRCC), and four (14%) concurred with two of the above types of RCC and/or clear cell (tubule) papillary tumor (Table 1).

Background kidney

Ninety-six percent (27/28) of these TC-RCC cases were found in kidneys with end-stage renal disease (Table 1).

Immunohistochemical profile

**Figure 2:**
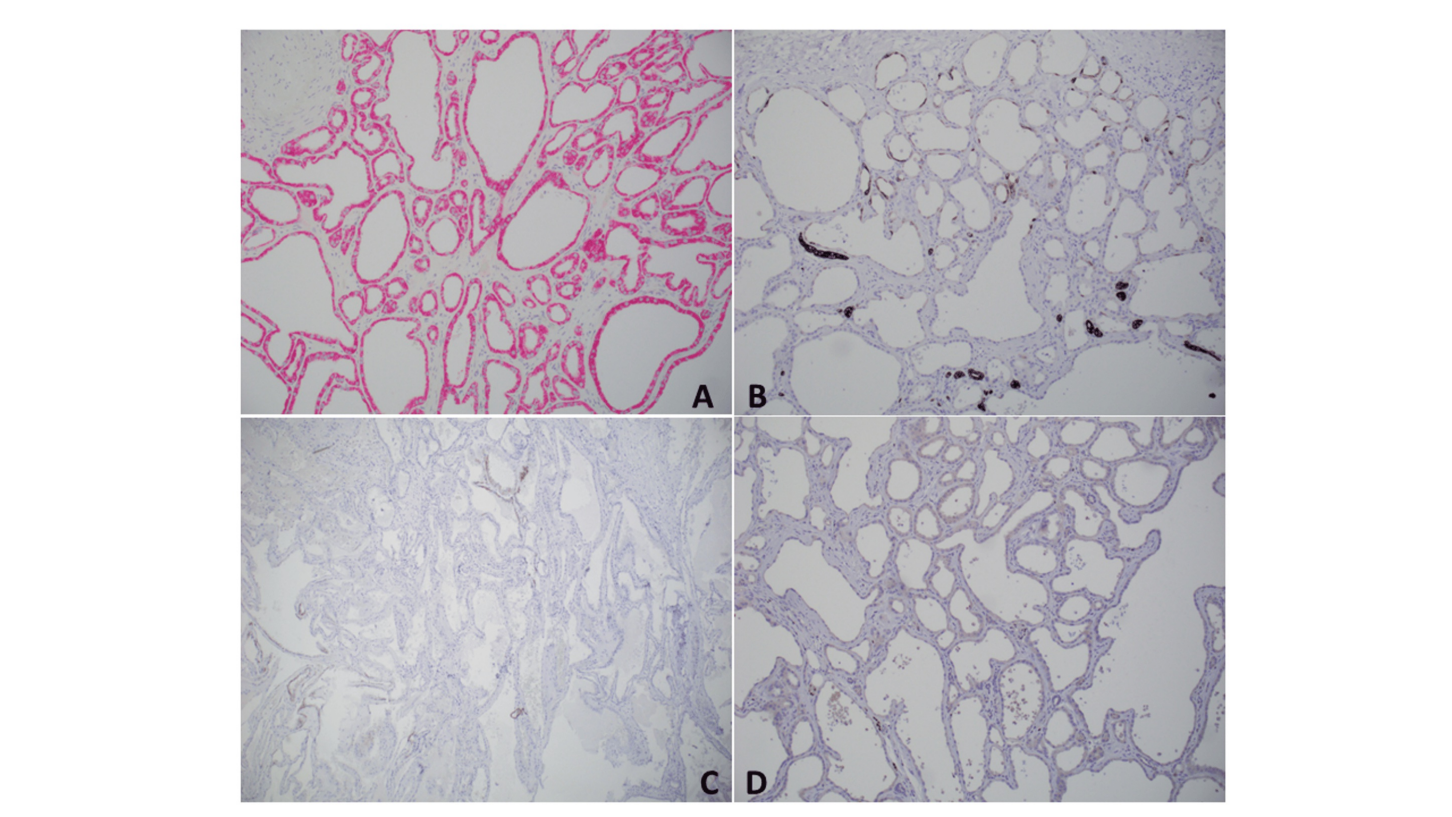
Immunohistochemical stains of the tubulocystic tumor (100 × magnification): A. AMACR positive; B. focal CK7 positive; C. rare/focal CAIX positive; D. GATA3 negative

The TC-RCCs for which IHC was performed were all consistently positive for AMACR, negative for GATA3, focal/rare cells positive or completely negative for CAIX, and focally positive or completely negative for CK7 (Figure 2 and Table 1).

Clinical course

Patients followed up from three months to seven years showed no metastasis or death from TC-RCC (Table 1).

An additional case of TC-RCC presenting as a retroperitoneal mass

**Figure 3:**
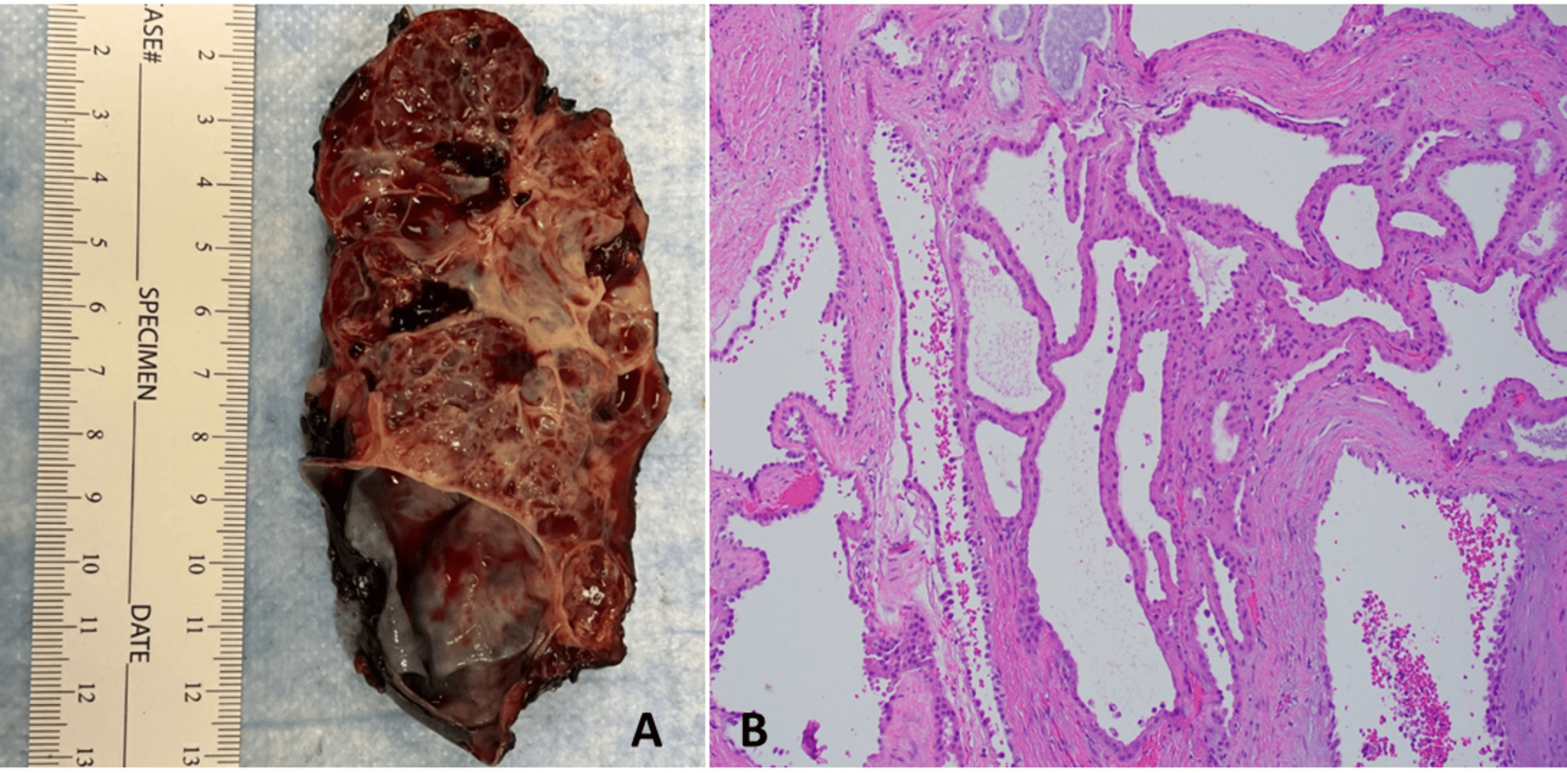
Retroperitoneal mass: A. Gross image shows a spongy appearance with variable-sized cysts; B. Hematoxylin and eosin section shows tubulocystic pattern with variable stroma; the tubulocystic lining cells exhibit eosinophilic cytoplasm and prominent nucleoli (100 × magnification).

This was the case of a 72-year-old female who presented with a 20-cm left retroperitoneal mass 20 years after left total nephrectomy for an unknown type of renal mass. Grossly, the retroperitoneal mass was cystic with a spongy appearance. A thorough microscopic examination of the mass revealed a pure and typical tubulocystic growth pattern as well as the cytomorphology of TC-RCC (Figure 3). Immunohistochemical staining showed the tumor cells to be negative for CK7, CK20, ALK, HMB45, melanin A, 2SC, TTF1, calretinin, ER, p53 (wild type), and WT1, and positive for AMACR, PAX8, AE1/AE3 (focal), CAIX (rare/focal), CD10, vimentin, AR, and fumarate hydratase. Ki67 was labeled <1% of tumor cells. A gene panel (648 genes) test performed at TEMPUS/xT confirmed its renal origin and showed only a NFE2L2 missense alteration. There was no evidence of metastatic disease anywhere. These findings supported the diagnosis of TC-RCC, presumably representing a recurrence of her incompletely resected TC-RCC, which grew and protruded into perinephric fat.

## Discussion

Tubulocystic growth patterns can be seen in many subtypes of renal neoplasm, including oncocytoma, acquired cystic disease-associated RCC, and fumarate hydratase-deficient RCC [4-6], and, depending on their associated tumor subtypes, their clinical courses differ greatly. It has recently been realized that some of the early reported TC-RCCs presenting with aggressive clinical courses likely represented other types of RCC with tubulocystic patterns unrelated to true TC-RCC, especially those cases with a prominent papillary, poorly differentiated, or sarcomatoid component [1, 7-10]. Those findings have promoted the WHO to redefine TC-RCC as a renal tumor with exclusive tubulocystic growth.

This study has shown that so-defined TC-RCCs were rare, predominantly seen in males, and located at the periphery of the kidney. They were usually small and multifocal and incidentally found almost exclusively in end-stage diseased kidneys.

End-stage kidney has been associated with up to 100 times increased risk of developing RCC [11-13] with a high frequency of association with ACD-RCC and other types of low-grade renal tumors, which have also been shown in this case series. 

The frequent concurrence of TC-RCC with papillary adenoma in end-stage kidney and their similar multifocality, indolent behavior, and shared chromosomal abnormalities (gains of chr 7 and 19 and loss of chr Y) [14-16] imply a similar pathogenesis for both tumors.

Given the above features of TC-RCC, similar to multilocular cystic neoplasm of low malignant potential (prior “multilocular cystic clear cell RCC”) and clear cell (tubulo)papillary renal cell tumor (prior “clear cell papillary RCC”), we propose that TC-RCC is better reclassified as tubulocystic renal tumor (tubulocystic adenoma when small and tubulocystic neoplasm of low malignant potential when large). It is also noteworthy that, although TC-RCC is an indolent tumor mostly confined to the kidney, it can grow and protrude deeply into perirenal fat due to its frequent peripheral location. Consequently, it can recur in the retroperitoneum if incompletely resected in a nephrectomy procedure for end-stage kidney or renal mass, as seen in one of the cases presented here in the results section.

This study is limited by the relatively small sample size, lack of robust clinical and additional molecular/genetic data, as well as the retrospective nature of the study design. Larger cohorts and more comprehensive studies are warranted to validate the results for adoption of the proposal.

## Conclusions

Twenty-eight cases of TC-RCC with pure tubulocystic pattern were studied for clinicopathological features. Most of the tumor presented with multifocality, peripheral location, and concurrence with papillary adenoma and/or other types of low-grade/indolent renal cell carcinoma in end-stage kidney, and all had indolent clinical courses. Given these features, we proposed to reclassify it as a tubulocystic renal cell tumor.
